# Ocular Comorbidities in Rosacea: A Case-Control Study Based on Seven Institutions

**DOI:** 10.3390/jcm10132897

**Published:** 2021-06-29

**Authors:** Yu Ri Woo, Minah Cho, Hyun Jeong Ju, Jung Min Bae, Sang Hyun Cho, Jeong Deuk Lee, Hei Sung Kim

**Affiliations:** 1 Department of Dermatology, Incheon St. Mary’s Hospital, The Catholic University of Korea, Seoul 06591, Korea; w1206@naver.com (Y.R.W.); macho_maria@naver.com (M.C.); drchos@yahoo.co.kr (S.H.C.); leejd@catholic.ac.kr (J.D.L.); 2 Department of Dermatology, St. Vincent’s Hospital, The Catholic University of Korea, Seoul 06591, Korea; hyd0116@naver.com; 3 Heal House Skin Clinic, Mesanro 24, Paldal-gu, Suwon 16461, Korea; jiminbae@gmail.com

**Keywords:** ocular comorbidities, rosacea, multi-institutional case-control study

## Abstract

Rosacea is a facial inflammatory dermatosis that is linked with various systemic illnesses. With regards to the eye, rosacea patients have been described to manifest ocular surface changes, such as blepharitis and conjunctivitis. However, studies that examine the association of rosacea with a wider array of ocular diseases are limited. Thus, our aim was to identify the range of ocular comorbidities in the Korean patient population and create a reference data set. A multi-institutional, case-control study was conducted, where 12,936 rosacea patients and an equal number of sex- and age-matched control subjects were extracted over a 12-year period. We were able to discover a notable association between rosacea and blepharitis (adjusted odds ratio (aOR) 3.44; 95% confidence interval, 2.71–4.36, *p* < 0.001), conjunctivitis (aOR 1.65; 95% CI, 1.50–1.82, *p* < 0.001), glaucoma (aOR 1.93; 95% CI, 1.70–2.20, *p* < 0.001), dry eye syndrome (aOR 1.89; 95% CI, 1.70–2.09, *p* < 0.001), and chalazion (aOR 3.26; 95% CI, 1.41–7.57, *p* = 0.006) from logistic regression analysis. Female subjects and individuals younger than 50 exclusively showed higher odds for chalazion. Our study suggests that ocular comorbidities (i.e., glaucoma, dry eye syndrome, and chalazion as well as blepharitis and conjunctivitis) are more prevalent among Koreans with rosacea. Clinicians should proactively check ocular symptoms in rosacea and employ joint care with an ophthalmologist in cases of need.

## 1. Introduction

Rosacea is a long-standing and recurring centrofacial skin condition with considerable burden of disease (i.e., profound negative influence on one’s self-esteem, quality of life, and well-being) [1,2,3,4,5,6,7,8].

The skin and eyes are equally derived from the embryonic surface ectoderm [9]. Not surprisingly, an association between rosacea and ocular surface changes has long been recognized, with interpalpebral conjunctival injection, lid margin telangiectasia, scleritis and sclerokeratitis, and spade-shaped corneal infiltrates being described as major features, and irregularity of the lid margin, collection of “honey crust” and scales on the eyelashes, and evaporative tear dysfunction representing secondary features in the 2017 standard classification of rosacea [10]. Rosacea has also been linked to corneal neovascularization and perforation, which can cause significant vision loss [11,12,13]. However, there are few comprehensive epidemiological studies on the ocular comorbidities of rosacea [14,15,16,17].

It is necessary that physicians caring for rosacea have evidence-based information on the ocular diseases to examine. Early detection of the ocular comorbidities will help manage the patient appropriately for a better outcome.

Rosacea is less apparent in darker skin types and with the lower degree of interest, data on ocular comorbidities in Asians are largely lacking [17]. Our study goal was to look into the chances of ocular comorbidities in Koreans with rosacea. Moreover, we examined the impact of sex and age on the interrelation of rosacea and different eye conditions.

The compulsory National health Insurance (NHI) system in South Korea allows researchers to perform nationwide population-based studies. However, certain skin conditions which are classified as “nonessential, cosmetic disorders” (i.e., androgenetic alopecia, melasma, acne, and rosacea) are not retrievable from the NHI database. Due to this limitation, we opted for a multi-center hospital database study.

## 2. Materials and Methods

### 2.1. Data Source

We conducted a seven-institutional cross-sectional study by securing the electronic medical records (EMR) data from the Catholic Medical Center (Incehon, Bucheon, Yeouido, Seoul, Eunpyeong, Uijeongbu, St. Mary’s hospital and St. Vincent’s Hospital) which is Korea’s largest healthcare network. Both subsidized and non-subsidized cases (based on the ICD-10 (International Classification of Disease, Tenth revision) diagnostic codes) are identified in our hospital database (the nU system). The data also provide information on patient sex, age, diagnosis, prescriptions, and the department and outpatient visitation dates [18]. This study was performed as a part of our rosacea-systemic comorbidity study.

### 2.2. Ethics

The Ethics Committee of the Catholic Medical Center reviewed and consented the study (XC19REDI0064) [18]. The study was performed under the principles of the Declaration of Helsinki.

### 2.3. Study Subjects

A population primarily diagnosed with rosacea, which includes other rosacea, rhinophyma, and rosacea unspecified (L71, L71.8, L71.1, L71.9), over a 12-year period (from 2007 to 2018) were selected from our nU system. To improve diagnostic accuracy, subjects were narrowed to those who visited the dermatology outpatient clinic at least twice with the diagnosis. In addition, individuals who failed to receive any treatment (i.e., lasers, oral and topical agents) for rosacea and subjects diagnosed two times or more with seborrheic dermatitis (L21), acne (L70), or systemic and cutaneous lupus erythematosus (M32 and L93 respectively) were excluded. The control population was sex- and age-matched individuals without rosacea who underwent appendectomy or hemorrhoidectomy during the exact same time span [18].

### 2.4. Concurrent Eye Conditions

Our outcome of interest was coexisting ocular conditions, including blepharitis (H01), conjunctivitis (H10), keratitis (H16), retinopathy (H35-H36), cataract (H25-H26), glaucoma (H40 and H42), herpesviral ocular disease (B00.50), chalazion (H00.1) and dry eye syndrome (H04.12). To be identified as having a particular eye condition, the patient had to be diagnosed at least twice from an ophthalmologist during the study period (2007 to 2018).

### 2.5. Statistical Analysis 

The 95% confidence intervals (CIs) and adjusted odds ratios (aORs) were calculated with the conditional logistic regression model. A subgroup analysis was also performed based on sex and age. We used the IBM SPSS version 26.0 (SPSS Inc., Chicago, IL, U.S.A.) for all analyses and *P* ≤ 0.05 was recognized as statistically significant.

## 3. Results

### 3.1. Basic Demographic Characteristics

This study was performed as a part of our rosacea-systemic comorbidity study [18]. A total of 12,936 rosacea patients and the exact same number of control subjects were included. As for patients with rosacea, the mean age was 47.4 years, with 34.0% being male. There was no statistical difference in sex and age between rosacea and the control population. Ocular diseases were present in 18.3% of rosacea patients and in 11.4% of the control population. The features of our study population are laid out in Table 1.

### 3.2. Interrelation of Rosacea and Ocular Disorders

The conditional logistic regression analysis on matched case-control pairs identified rosacea to be appreciably associated with various ocular diseases (blepharitis (adjusted odds ratio (aOR) 3.44; 95% confidence interval, 2.71–4.36, *p* < 0.001), conjunctivitis (aOR 1.65; 95% CI, 1.50–1.82, *p* < 0.001), glaucoma (aOR 1.93; 95% CI, 1.70–2.20, *p* < 0.001), dry eye syndrome (aOR 1.89; 95% CI, 1.70–2.09, *p* < 0.001), and chalazion (aOR 3.26; 95% CI, 1.41–7.57, *p* = 0.006)) (Table 2).

### 3.3. Subgroup Analysis by Sex 

When stratified by sex, women with rosacea showed significant association with a number of ocular diseases (blepharitis (aOR 3.17; 95% CI, 2.40–4.18, *p* < 0.001), conjunctivitis (aOR 1.63; 95% CI, 1.45–1.82, *p* < 0.001), glaucoma (aOR 2.14; 95% CI, 1.82–2.52, *p* < 0.001), dry eye syndrome (aOR 1.94; 95% CI, 1.72–2.19, *p* < 0.001), and chalazion (aOR 3.93; 95% CI, 1.11–13.84, *p* = 0.03)) (Table 3).

Male subjects were notably associated with a variety of ocular diseases (blepharitis (aOR 4.22; 95% CI, 2.63–6.77, *p* < 0.001), conjunctivitis (aOR 1.71; 95% CI, 1.43–2.05, *p* < 0.001), glaucoma (aOR 1.58; 95% CI, 1.27–1.97, *P* < 0.001), and dry eye syndrome (aOR 1.75; 95% CI, 1.45–2.12, *P* < 0.001)) (Table 3).

### 3.4. Subgroup Analysis with Age 

With the median age of our study population being 50 years, we chose 50 as the cutoff value for our two subgroups (“50 years and higher” vs. “Under 50”). Upon subgrouppanalysis with age, patients 50 and higher were appreciably associated with a variety of ocular diseases (blepharitis (aOR 3.00; 95% CI, 2.27–3.97, *p* < 0.001), conjunctivitis (aOR 1.43; 95% CI, 1.27–1.61, *p* < 0.001), glaucoma (aOR 1.48; 95% CI, 1.26–1.73, *p* < 0.001), and dry eye syndrome (aOR 2.16; 95% CI, 1.89–2.46, *p* < 0.001)) (Table 3).

Subjects under 50 were linked with a number of ocular diseases (blepharitis (aOR 4.51; 95% CI, 2.82–7.23, *P* < 0.001), conjunctivitis (aOR 2.14; 95% CI, 1.80–2.55, *p* < 0.001), glaucoma (aOR 3.53; 95% CI, 2.66–4.69, *p* < 0.001), dry eye syndrome (aOR 1.46; 95% CI, 1.25–1.71, *p* < 0.001), and chalazion (aOR 3.40; 95% CI, 1.14–10.14, *p* = 0.02)) (Table 3).

## 4. Discussion

Our findings reveal that Koreans with rosacea are prone to experience various eye complications compared to the control population. This is in accordance with our previous report where we identified that Koreans with rosacea have a higher risk of systemic comorbidity [18]. Despite being a single study [18], we have separated the eye conditions from other systemic comorbidities on grounds of its significance in rosacea. The greatest increase among the different ophthalmic comorbidities was blepharitis, followed by chalazion, glaucoma, dry eye syndrome, and conjunctivitis.

The etiology of the findings is likely multifactorial, involving inflammation, barrier disruption, and microbes [15,16,17,19]. Blepharitis, an inflammation of the eyelids, is categorized as anterior or posterior depending on the anatomic location relative to the lash margin. Anterior blepharitis is commonly due to infection (i.e., staphylococcus, Demodex mites), and posterior blepharitis is secondary to blockage of the meibomian oil glands [20]. It is most likely that the immune dysregulation in rosacea patients, accompanied by barrier disruption and trans-epidermal water loss, promotes eyelid skin inflammation. Of note, inflammation (and the release of pro-inflammatory cytokines and enzyme) of the eyelid can cause meibomian gland dysfunction [19].

Studies have shown that Demodex mite (*D. brevis* and *folliculorum*) density is increased in the skin and eyelid/eyelashes of patients with rosacea [21,22,23]. In addition, significant correlation was found between facial rosacea, eyelid inflammation and Demodex eye infestation with serum immunoreactivity to Bacillus proteins [24,25]. Demodex mites are believed to induce acute blepharitis and conjunctivitis (thereby inducing inflammation) by causing skin disruption [26], immune evasion [27,28] and bacterial (i.e., staphylococci and sterptococci) transfer [29,30]. In addition, *D. brevis* can physically block the meibomian gland, inducing chalazion (a lump in the eyelid caused by obstruction and inflammation of the meibomian gland), glandular inflammation, and tear lipid paucity [19,31].

Meibomian gland blockage and inflammation are important components of meibomian gland dysfunction in rosacea. Meibomian gland dysfunction changes the meibum quality and quantity, which can cause dry eye symptoms in affected individuals [32]. Often, dry eye has no long-term sequelae. However, if the situation is grave and left unattended, corneal damage and complications follow, which may lead to vision loss or impairment [17]. While oral doxycycline and isotretinoin are often prescribed in rosacea, the deleterious effect of isotretinoin on the meibomian gland is well known [33,34]. Their ocular effect was compared to find improvement in meibomian gland function (increased tear film stability) and ocular symptoms with doxycycline [35]. With the administration of low-dose isotretinoin, a number of patients reported worsening of the meibomian gland secretion and eye symptoms [35], which suggests that isotretinoin should be used with caution.

Interestingly, we observed an increased risk of glaucoma, which has rarely been mentioned in association with rosacea. Glaucoma is a group of eye diseases which result in damage to the optic nerve and cause vision loss [20]. Multiple studies have connected glaucoma to corticosteroids application on the face, from direct contact and absorption by ocular tissues [36,37,38]. Systemic steroids consumed for more than 2 months has also been linked with a dramatic rise in intra-ocular pressure [39]. Given that glaucoma is often without any symptom until it is fully blown, early detection is crucial. Regular ophthalmic screening is necessary for those with a history of repetitious and chronic steroid use, and family history of glaucoma [40].

Our study has limitations. First, we were not able to fully identify the disease severity and subtype of rosacea, as well as the risk factors (i.e., complete medication history on both prescription and OTC (over-the-counter) drugs) from our hospital database. The confounding effect of these factors are to be examined in future studies to confirm our findings. Second, there is a possibility that steroid-induced rosacea cases were included in our study population since they share the same diagnostic code and treatment with rosacea. This, together with the adopted health care setting (i.e., secondary and tertiary referral hospitals) may have affected the odds of the comorbid eye conditions. Additionally, a clear temporal relationship between the development of rosacea and the ocular comorbidity is absent owing to the retrospective nature of the study.

In conclusion, this is the very first study to examine the ocular comorbidities of rosacea in Koreans. We discovered a high burden of ocular comorbidity where more of the individuals with rosacea had blepharitis, chalazion, glaucoma, dry eye syndrome, and conjunctivitis. Notably, higher odds for chalazion were exclusive in female subjects and individuals under 50 when stratified by sex and age. Here, we would like to clarify that many of the ocular comorbidities that we identified are not necessarily specific to rosacea. Nevertheless, our findings highlight the need for collaboration between physicians involved in rosacea and ophthalmologists. Despite the potential risk of vision loss, the importance of a regular ophthalmologic examination is often ignored in rosacea. Through improved awareness and early intervention, vision-threatening complications can be very much prevented. We also encourage further studies on rosacea and its ocular comorbidities from different countries which are necessary to better understand and manage the disease.

## Figures and Tables

**Table 1:** Study population characteristics.

	Rosacea Patients (N = 12,936)	Controls without Rosacea (N = 12,936)
Sex, N (%)		
Female	8540 (66.0)	8540 (66.0)
Male	4396 (34.0)	4396 (34.0)
Age, years, N (%)	47.4 ± 0.13	48.4 ± 0.13
<20	679 (5.3)	679 (5.3)
20–39	3363 (26.0)	3363 (26.0)
40–59	6252 (48.3)	6252 (48.3)
60–79	2538 (19.6)	(19.6)
>80	104 (0.80)	104 (0.80)
Ocular disorder		
Present	2371 (18.3)	1472 (11.4)
Absent	10,565 (81.7)	11,464 (88.6)

Data are presented in number (%).

**Table 2:** Interrelation of rosacea and ocular conditions.

Variables	Number of Cases		OR	95% CI	*p*-Value	aOR*	95% CI	*p*-Value
	Rosacea	Control						
Ocular Disorder								
Blepharitis	360	81	3.34	2.63–4.23	<0.001	3.44	2.71–4.36	<0.001
Conjunctivitis	1136	521	1.64	1.49–1.81	<0.001	1.65	1.50–1.82	<0.001
Keratitis	358	383	0.58	0.51–0.67	0.05	0.68	0.58–0.78	0.06
Glaucoma	735	288	1.91	1.67–2.17	<0.001	1.93	1.70–2.20	<0.001
Retinopathy	160	216	0.83	0.69–0.9	0.04	0.88	0.73–1.06	0.19
Cataract	91	65	1.05	0.76–1.44	0.75	1.27	0.92–1.75	0.13
Dry eye syndrome	1099	645	1.76	1.60–1.95	<0.001	1.89	1.70–2.09	<0.001
Herpesviral ocular disease	12	10	1.20	0.51–2.77	0.67	1.22	0.52–2.88	0.63
Chalazion	25	7	3.57	1.54–8.27	0.003	3.26	1.41–7.57	0.006

OR, odds ratio; CI, confidence interval; aOR*, adjusted odds ratio, adjusted by sex and age.

**Table 3:** Subgroup Analyses of the interrelation of rosacea and ocular conditions by age and sex.

Variables	Sex						Age					
	Male			Female			<50			≥50		
	aOR*	95% CI	*p*-Value	aOR*	95% CI	*p*-Value	aOR*	95% CI	*p*-Value	aOR*	95% CI	*p*-Value
**Ocular Conditions**												
Blepharitis	4.22	2.63–6.77	<0.001	3.17	2.40–4.18	<0.001	4.51	2.82–7.23	<0.001	3.00	2.27–3.97	<0.001
Conjunctivitis	1.71	1.43–2.05	<0.001	1.63	1.45–1.82	<0.001	2.14	1.80–2.55	<0.001	1.43	1.27–1.61	<0.001
Keratitis	0.77	0.58–1.00	0.05	0.70	0.59–0.84	0.08	0.70	0.60–0.81	0.06	0.59	0.49–0.71	0.07
Glaucoma	1.58	1.27–1.97	<0.001	2.14	1.82–2.52	<0.001	3.53	2.66–4.69	<0.001	1.48	1.26–1.73	<0.001
Retinopathy	0.79	0.57–1.10	0.16	0.92	0.73–1.15	0.49	1.29	0.89–1.86	0.17	0.76	0.61–0.94	0.15
Cataract	1.25	0.75–2.06	0.37	1.28	0.84–1.95	0.23	1.03	0.74–1.41	0.85	1.36	0.91–1.92	0.07
Dry eye syndrome	1.75	1.45–2.12	<0.001	1.94	1.72–2.19	<0.001	1.46	1.25–1.71	<0.001	2.16	1.89–2.46	<0.001
Herpesviral ocular disease	0.81	0.23–2.89	0.75	1.68	0.53–5.35	0.37	1.12	0.31–4.01	0.85	1.22	0.39–3.79	0.72
Chalazion	2.79	0.89–8.70	0.07	3.93	1.11–13.84	0.03	3.40	1.14–10.14	0.02	3.13	0.83–11.82	0.09

aOR*, adjusted odds ratio, adjusted by age and sex; CI, confidence interval.
